# Risk Factors Associated With the Number of Symptoms and Distress Caused by Genitourinary Syndrome of Menopause in Taiwanese Women

**DOI:** 10.1002/kjm2.70143

**Published:** 2025-12-17

**Authors:** Shu‐Fang Su, Chiu‐Lin Wang, Shu‐Yuan Lin

**Affiliations:** ^1^ Department of Nursing Kaohsiung Show Chwan Memorial Hospital Kaohsiung City Taiwan; ^2^ School of Nursing Kaohsiung Medical University Kaohsiung Taiwan; ^3^ Department of Obstetrics and Gynecology Kaohsiung Municipal Siaogang Hospital, Kaohsiung Medical University Kaohsiung Taiwan; ^4^ Department of Obstetrics and Gynecology Kaohsiung Medical University Hospital, Kaohsiung Medical University Kaohsiung Taiwan; ^5^ Graduate Institute of Medicine, College of Medicine, Kaohsiung Medical University Kaohsiung Taiwan; ^6^ Department of Medical Research Kaohsiung Medical University Hospital Kaohsiung Taiwan

**Keywords:** distress, genitourinary syndrome of menopause, health‐seeking behaviors, menopausal symptoms

## Abstract

A high percentage of women who undergo the transition to postmenopause experience both menopausal symptoms and genitourinary syndrome of menopause (GSM). However, GSM is often underdiagnosed. This research aims to identify risk factors that may influence the number of GSM symptoms and whether they cause distress in Taiwanese women > 45 years of age who experienced at least one GSM symptom. Data were collected using a self‐developed questionnaire, the Taiwanese Greene Climacteric Scale, and the Genitourinary Symptoms Scale. Participants reported a low frequency and low level of distress related to menopause symptoms (14.33 ± 9.12; 10.75 ± 10.01, respectively) with an average of 3.64 genitourinary symptoms experienced. The majority of participants (59.7%) reported experiencing distress regarding GSM. Multivariate analyses indicated that the distress level of menopause symptoms (*p* < 0.001), distress caused by genitourinary symptoms (*p* < 0.001), BMI > 24.0 kg/m^2^ (*p* = 0.034) and seeking medical consultation for incontinence (*p* < 0.001) were significantly associated with the number of genitourinary symptoms. Notably, a higher level of distress regarding menopause symptoms (*p* = 0.022), a greater number of genitourinary symptoms (*p* < 0.001), and having sought medical consultation due to genitourinary symptoms (*p* = 0.007) were significantly associated with experiencing distress caused by genitourinary symptoms. This study highlights the importance of patient education, proactive discussions, and addressing barriers to healthcare and cultural factors in GSM management. Healthcare professionals might consider providing additional assessments for GSM in older women who are admitted to the hospital for other diseases and highlight the distress caused by genitourinary symptoms, as GSM is frequently underreported yet treatable.

## 1 Introduction

Menopause, defined retrospectively after 12 consecutive months of amenorrhea [1, 2, 3], often begins at age 50–52 [1, 4, 5]. However, around the age of 47, women may experience perimenopause, characterized by increased variability in menstrual cycle length that continues through the transition for five to eight years [6]. The World Health Organization estimates that by 2030, 1.2 billion women over 50 years of age will experience menopause [4]. Menopause symptoms including somatic, vasomotor, and psychological symptoms are common during this transition and are closely linked to the physiological changes associated with aging [1, 3]. Somatic symptoms, including arthralgia, myalgia, headaches, and dizziness, affect 52%–96% of menopausal women. Psychological symptoms such as sleep disturbances, anxiety, and depression affect 83%–91% of women during menopause [7], and vasomotor symptoms such as hot flashes are experienced by approximately 80% [7, 8, 9]. A study using data from 20,882 Taiwanese women registered in the nationwide toll‐free menopause consultation hotline database demonstrated that the most frequent menopause symptoms were memory loss (66.9%), fatigue (66.5%), insomnia (59.6%), depressed mood (58.5%), and back pain (58.2%) [10], revealing a broad spectrum of symptoms that impact women's health.

Genitourinary syndrome of menopause (GSM) occurs in 50%–84% of menopausal women. Caused by the decrease in estrogen levels during the menopausal transition [11, 12, 13, 14], GSM comprises genital symptoms (dryness, burning, itching, irritation, bleeding), sexual symptoms (dyspareunia and other sexual dysfunctions), and urinary symptoms (dysuria, frequency, urgency, recurrent urinary tract infections) [11, 15]. Menopausal women seek gynecological examinations for symptoms including vaginal dryness (100%), dyspareunia (78%), burning (57%), itching (57%), and dysuria (32%), which occur 1–6 years after menopause [14]. Among Taiwanese menopausal women, the prevalence of vaginal dryness increased by approximately 20%, from 37.6% in the premenopausal stage to 56.6% in the postmenopausal stage. Similarly, the prevalence of dyspareunia increased by approximately 15%, from 31.9% to 45.3% between the premenopausal and postmenopausal stages [10]. Unresolved genitourinary symptoms have negative effects on women's daily activities, sexual life, and health‐related quality of life [16].

Several risk factors for GSM have been identified. A cross‐sectional study conducted in middle‐aged and older women in the Beijing community revealed that older age (ages 55–64), (odds ratio [OR], 8.63), menopause (OR, 2.20), and fecal incontinence (OR, 1.31) were associated with a higher risk for GSM [15]. Additionally, a study of a cohort of Greek peri‐ and postmenopausal women also showed that age, weight, body mass index (BMI), and the number of pregnancies and abortions were associated with the risk of lower urinary tract symptoms, which is one component of GSM [17]. GSM is often underdiagnosed due to insufficient screening and patient reluctance to discuss symptoms. The most common reason for not discussing their GSM was because they accepted it as part of the natural aging process [5]. However, GSM symptoms are more prevalent and severe in postmenopausal women than in perimenopausal women, with severity increasing with time since menopause rather than age [18]. As reported in the Pan‐Asia REVIVE survey [19], only 35% of postmenopausal women with GSM symptoms were aware of the condition, with most first learning about GSM through their physician (32%). Additionally, only 25% of women had discussed their GSM symptoms with a healthcare professional, and 24% of women had ever used treatment for their symptoms [19]. Similarly, only 10%–15% of Taiwanese menopausal women reported seeking medical consultation [20]. Barriers such as cultural stigma and lack of awareness were found to lead to undertreatment in many Asian countries [19].

Understanding the risk factors for GSM will reveal barriers to health‐seeking behaviors in women and increase healthcare professionals' knowledge to develop effective interventions. Therefore, to improve the management of GSM, this study aims to determine whether individual characteristics, health‐seeking behaviors, and menopausal symptoms influence the number of symptoms and distress level of GSM in Taiwanese women.

## 2 Materials and Methods

### 2.1 Study Design and Sample

This study was a cross‐sectional questionnaire survey conducted from May 2022 to October 2023. Participants were invited by the primary researcher at the obstetrics and gynecology outpatient clinic and the physical examination center at a regional hospital in southern Taiwan. Women who were over 45 years of age, experiencing at least one symptom of GSM (such as vaginal dryness, irritation, dyspareunia, urinary frequency, urgency, incontinence, or urinary tract or vaginal infections), had irregular menstruation or amenorrhea, and had spouses or intimate partners were recruited. Women who had menopause due to a medical regimen, were taking birth control pills, underwent gynecological surgery such as oophorectomy or hysterectomy, or had a diagnosis of breast cancer were excluded from the study. Questionnaires were completed by 240 eligible women, 9 of who experienced no menopausal symptoms were excluded. A total of 231 women were included in the analysis, with a 96% completion rate.

### 2.2 Instruments

This study used data from a self‐developed questionnaire addressing individual characteristics and health‐seeking behaviors, the Taiwanese version of the Greene Climacteric Scale (T‐GCS) [21], and the Genitourinary Symptoms Scale (GSS) [16].

- The self‐developed questionnaire had two parts; the first part included items regarding individual characteristics such as age, BMI, educational level, employment status, number of children, diagnosis of chronic diseases, age at menopause, and menopause status. The second part inquired about health‐seeking behaviors, including seeking medical consultation regarding menopause, genitourinary symptoms or incontinence; taking Chinese medicine; taking medication, hormone therapy, or nutritional supplements; and seeking health information about menopause or genitourinary symptoms.

#### 2.2.1 Taiwanese Version of the Greene Climacteric Scale (T‐GCS)

T‐GCS was translated from the Greene Climacteric Scale to assess women's menopausal symptoms [21]. The T‐GCS comprises 21 items in four categories: psychological symptoms (11 items), somatic symptoms (7 items), vasomotor symptoms (2 items), and sexual function (1 item) [22, 23]. The T‐GCS has a modified response scale assessing two parameters: the frequency of symptom occurrence and the degree of distress, both rated on a 4‐point Likert scale. The frequency of symptom occurrence ranged from 0 to 3, from “absent”(0 points) to “always” (3 points); the degree of distress ranged from “not at all” (0 points) to “severe” (3 points). The content validity index (CVI) reached 0.92, and Cronbach's *α* coefficients for reliability were 0.84 and 0.89 for the scales of frequency of symptom occurrence and symptom distress, respectively [21]. In the present study, Cronbach's *α* coefficients were 0.88 and 0.93 for the scales of frequency of symptom occurrence and symptom distress total scale, respectively.

#### 2.2.2 The Genitourinary Symptoms Scale (GSS)

The GSS was developed to assess participants' experiences with 11 common genitourinary symptoms, including urinary leakage, frequent voiding, urge voiding, nighttime voiding, interruptions during voiding, required assistance to start or continue urinating, residual sensation or incomplete bladder‐empty, a weak urine stream, vaginal dryness, vaginal itching, and difficulties during sexual intercourse [16]. Each symptom was responded to in one of two options: present (1 point) or absent (0 points). The total score for the genitourinary symptoms ranged from 0 to 11, with higher scores reflecting more symptoms experienced. One item inquiring “To what degree were you bothered by the genitourinary symptoms in the last 12 months?” offered a modified response range of 0–10 to better determine the degree of distress. Four levels of distress were defined according to the scores: not at all (0), mild (1–3), moderate (4–7), and severe (8–10). The CVI was 1.0, and the Kuder–Richardson Formula 21 (KR‐21) coefficient for internal consistency was 0.73 [16]. The test‐retest reliability shown by Phi correlations ranged from 0.74 to 1.00 [16]. In this study, the KR‐21 coefficient was 0.72. Questionnaire results are shown in Table S1.

### 2.3 Data Collection and Ethical Consideration

This study was approved by the Institutional Review Board of the researchers' Hospital (KMUHIRB‐E(II)‐xxxx0066). The primary researcher approached potential participants and explained the study's plan and the participants' rights. Voluntariness, confidentiality, privacy, and no increase in daily risks were maintained. Participants filled out the questionnaires by themselves after signing the informed consent. For women who had problems reading or writing, face‐to‐face interviews were conducted to help them to complete the questionnaires. Participants spent 15–20 min answering all of the questionnaires.

### 2.4 Data Analysis

After checking and ensuring data accuracy, we conducted the statistical analysis using IBM SPSS 26.0. Distributions of variables in individual characteristics, health‐seeking behaviors, menopausal symptoms, and genitourinary symptoms are indicated by frequency/percentage for categorical variables and mean/standard deviation (SD) for continuous variables. Spearman correlations, a rank‐order correlation, and a non‐parametric version of the Pearson correlation without normal distribution assumption were used to investigate the relationships between individual characteristics, health‐seeking behaviors, and menopausal symptoms and the occurrence and severity of genitourinary symptoms. We treated the number of genitourinary symptoms as ordered categories, and assumed that the effect of each predictor is consistent across all levels of genitourinary symptoms numbers (i.e., proportional odds assumption). As a result, factors associated with the number of genitourinary symptoms were identified by ordinal logistic regression analysis. An odds ratio greater than 1 indicates that higher values of the predictor are associated with increased odds of being in a higher category of genitourinary symptoms. Furthermore, binary logistic regression analysis was used to identify predictors for whether participants experienced genitourinary symptom distress. All statistical tests were set at a significance level below 0.05.

## 3 Results

### 3.1 Participant Characteristics, Health‐Seeking Behaviors, and Characteristics of Menopause and Genitourinary Symptoms

The mean age of study participants was 56.53 years (SD, 4.31), and the mean age at menopause was 51.64 years (SD, 2.91). Elevated BMI (> 24.0 kg/m^2^) was present in 40.2% of participants, and the most frequent comorbidities were hypertension (31.3%) followed by arthritis (19.4%). The majority of participants (79.2%) were postmenopausal. Most of the participants took no medication (94.4%) or hormone therapy (81.8%) for their menopause status. While 90.9% of the participants had sought health information from resources such as the web, friends, or professionals, only 33.3% and 33.8% had sought medical consultation for genitourinary and menopause symptoms, respectively. We observed a low frequency of menopause symptoms (14.33 ± 9.12) and a low level of distress regarding these symptoms (10.75 ± 10.01) among participants. The mean number of genitourinary symptoms experienced was 3.64 (SD, 2.64) and these symptoms were perceived as distressing in 59.7% of the women (Table 1).

**TABLE 1:** Participant characteristics and health‐seeking behaviors (*N* = 231).

	*n*	%	M ± SD
Age (years)			56.53 ± 4.31
Age at menopause			51.64 ± 2.91
Number of children			
0–2	163	70.6	
3–5	68	29.4	
BMI (kg/m^2^)			
< 18.5	11	4.8	
18.5–24.0	127	55.0	
≥ 24.0	93	40.2	
Educational level			
Less than high school	40	17.3	
High school	103	44.6	
College or above	88	38.1	
Employment			
Employed	108	46.8	
Retired/unemployed	123	53.2	
Comorbidity (*n* = 211)			
Arthritis	41	19.4	
Cardiovascular disease	16	7.6	
Hypertension	66	31.3	
Diabetes	23	10.9	
Cancer	15	7.1	
Gynecological disease	13	6.2	
Depression	7	3.3	
Other	30	14.2	
Menopause status			
Perimenopausal	48	20.8	
Postmenopausal	183	79.2	
Seeking medical consultation for menopause			
No	153	66.2	
Yes	78	33.8	
Seeking medical consultation for genitourinary symptoms			
No	154	66.7	
Yes	77	33.3	
Seeking medical consultation for incontinence			
No	184	79.7	
Yes	47	20.3	
Taking medication			
No	218	94.4	
Yes	13	5.6	
Taking nutritional supplements			
No	110	7.6	
Yes	121	52.4	
Hormone therapy			
No	189	81.8	
Yes	42	18.2	
Taking Chinese medicine			
No	206	89.2	
Yes	25	10.8	
Seeking health information			
No	21	9.1	
Yes	210	90.9	
Frequency of menopause symptoms			14.33 ± 9.12
Distress level of menopause symptoms			10.75 ± 10.01
Number of genitourinary symptoms			3.64 ± 2.64
Distress regarding genitourinary symptoms			
No	93	40.3	
Yes	138	59.7	

Abbreviations: M, mean; SD, standard deviation.

### 3.2 Correlation Analysis of Possible Risk Factors

The correlation analysis results are presented in Table 2. Both the frequency and distress level of menopause symptoms correlated positively with the number of genitourinary symptoms and whether these symptoms caused distress (*r*, 0.39–0.84; *p* < 0.001). Seeking medical consultation regarding menopause, genitourinary symptoms or incontinence, and hormone therapy correlated positively with both the frequency and distress level of menopause symptoms (*r*, 0.13–0.36; all *p* < 0.05). Similarly, seeking medical consultation regarding menopause, genitourinary symptoms or incontinence, and hormone therapy correlated positively with the number of genitourinary symptoms and whether these symptoms caused distress (*r*, 0.16–0.39; all *p* < 0.05). The number of children and BMI correlated weakly with either menopause or genitourinary symptoms (*r*, 0.02–0.15).

**TABLE 2:** Spearman correlations between individual characteristics, health‐seeking behaviors, menopause, and genitourinary symptoms.

	Correlation matrix *r (p)*									
	1	2	3	4	5	6	7	8	9	10
Frequency of menopause symptoms	1									
2 Distress level of menopause symptoms	0.84**	1								
	(< 0.001)									
3 Number of genitourinary symptoms	0.43**	0.40**	1							
	(< 0.001)	(< 0.001)								
4 Distress caused by genitourinary symptoms (No/Yes)	0.39**	0.39**	0.65**	1						
	(< 0.001)	(< 0.001)	(< 0.001)							
5 Number of children	0.02	−0.06	0.09	0.12	1					
	(0.790)	(0.345)	(0.180)	(0.067)						
6 BMI	0.11	0.04	0.15*	0.04	0.21**	1				
	(0.101)	(0.543)	(0.019)	(0.506)	(0.002)					
7 Sought medical consultation for menopause symptoms	0.36**	0.32**	0.16*	0.25**	−0.02	−0.05	1			
	(< 0.001)	(< 0.001)	(0.013)	(< 0.001)	(0.743)	(0.498)				
8 Sought medical consultation for genitourinary symptoms	0.18**	0.16*	0.23**	0.34**	−0.01	−0.07	0.17**	1		
	(0.005)	(0.017)	(< 0.001)	(< 0.001)	(0.849)	(0.257)	(0.008)			
9 Sought medical consultation for incontinence	0.13*	0.15*	0.39**	0.26**	−0.01	0.11	0.07	0.24**	1	
	(0.049)	(0.025)	(< 0.001)	(< 0.001)	(0.886)	(0.091)	(0.281)	(< 0.001)		
10 Hormone therapy	0.20**	0.20**	0.17**	0.25**	−0.003	0.03	0.42**	0.21**	0.07	1
	(0.002)	(0.002)	(0.009)	(< 0.001)	(0.968)	(0.706)	(< 0.001)	(0.001)	(0.300)	

*Note*: *p < 0.05; **p < 0.01.

### 3.3 Risk Factors for the Number and Distress Caused by Genitourinary Symptoms

Results of prediction models for the number of genitourinary symptoms are shown in Table 3. Univariate analyses showed that all predictors were significantly associated with the number of genitourinary symptoms (OR = 1.087–19.12, all *p* < 0.05). However, the c‐index ranged from 0.530 to 0.706, suggesting low to mild discriminatory ability for these factors. After including all predictors in the multivariate analyses, the distress level of menopause symptoms (aOR, 1.051; 95% CI, 1.024–1.079; *p* < 0.001), distress caused by genitourinary symptoms (aOR, 14.35; 95% CI, 7.474–27.56; *p* < 0.001), BMI > 24.0 kg/m^2^ (aOR, 1.688; 95% CI, 1.040–2.740; *p* = 0.034) and seeking medical consultation for incontinence (aOR, 4.772; 95% CI, 2.541–8.962; *p* < 0.001) remained statistically significant. The Nagelkerke R^2^ was 0.511 and the c‐index was 0.785 in the multivariate model.

**TABLE 3:** Prediction of risk factors for the number of genitourinary symptoms.

	Univariate ordinal logistic model			Multiple ordinal logistic model^a^	
	OR (95% CI)	*p*	c‐index	aOR (95% CI)	*p*
Frequency of menopause symptoms^b^	1.105 (1.075–1.136)	< 0.001**	0.667		
Distress level of menopause symptoms	1.087 (1.061–1.114)	< 0.001**	0.652	1.051 (1.024–1.079)	< 0.001**
Distress caused by genitourinary symptoms	19.12 (10.50–34.82)	< 0.001**	0.706	14.35 (7.474–27.56)	< 0.001**
Number of children	1.330 (1.016–1.741)	0.038*	0.530	1.123 (0.849–1.486)	0.417
BMI > 24.0	1.741 (1.095–2.767)	0.019*	0.549	1.688 (1.040–2.740)	0.034*
Sought medical consultation for menopause symptoms^c^	1.815 (1.122–2.935)	0.015*	0.550	0.884 (0.507–1.543)	0.6650
Sought medical consultation for genitourinary symptoms^c^	2.330 (1.431–3.794)	< 0.001**	0.571	0.969 (0.570–1.648)	0.909
Sought medical consultation for incontinence^c^	6.699 (3.653–12.29)	< 0.001**	0.603	4.772 (2.541–8.962)	< 0.001**
Hormone therapy^c^	2.081 (1.154–3.752)	0.015*	0.543	0.842 (0.433–1.638)	0.612

*Note*: *p < 0.05; **p < 0.01.

^a^ Logistic regression model: all women (*N* = 231); Likelihood ratio test, χ^2^ = 163; *p* < 0.001; Nagelkerke R^2^, 0.511; c‐index = 0.785.

^b^ ^“^Frequency of menopause symptoms” was not included in the multivariate model due to a high correlation with “Distress of menopause symptoms” (Spearman *r*, 0.84).

^c^ Reference groups: Sought medical consultation for menopause symptom = No; Sought medical consultation for genitourinary symptom = No; Sought medical consultation for incontinence = No; Hormone therapy = No.

Results of prediction models for distress caused by genitourinary symptoms are shown in Table 4. All selected covariates except BMI were significant risk factors of distress caused by genitourinary symptoms (OR, 1.118–5.322; all *p* < 0.05). The number of genitourinary symptoms moderately associated with whether genitourinary symptoms caused distress (c‐index, 0.878). A higher level of distress from menopause symptoms (aOR, 1.067; 95% CI, 1.009–1.129; *p* = 0.022) and a greater number of genitourinary symptoms (aOR, 2.233; 95% CI, 1.723–2.951; *p* < 0.001) were associated with having distress caused by genitourinary symptoms. Furthermore, individuals who had sought medical consultation due to genitourinary symptoms were 3.4 times more likely to have distress caused by genitourinary symptoms than their counterparts (aOR, 3.401; 95% CI, 1.405–8.232; *p* = 0.007). In the multivariate model, the c‐index was 0.917 and the Nagelkerke R^2^ was 0.626.

**TABLE 4:** Prediction models for distress caused by genitourinary symptoms^a^.

	Distress caused by genitourinary symptoms^d^		Univariate logistic model			Multiple logistic model	
	No (*n* = 93)	Yes (*n* = 138)	OR (95% CI)	*p*	c‐index	OR (95% CI)	*p*
Frequency of menopause symptoms^b^	10.6 ± 6.7	17.1 ± 9.5	1.121 (1.075–1.170)	< 0.001**	0.731		
Distress level of menopause symptoms	6.2 ± 6.4	13.8 ± 10.0	1.118 (1.073–1.165)	< 0.001**	0.730	1.067 (1.009–1.129)	0.022*
Number of genitourinary symptoms	1.7 ± 1.6	4.9 ± 2.4	2.432 (1.922–3.076)	< 0.001**	0.878	2.233 (1.723–2.894)	< 0.001**
Number of children	2.0 ± 0.9	2.3 ± 0.8	1.418 (1.026–1.959)	0.034*	0.564	1.762 (1.053–2.951)	0.031*
BMI > 24.0	35 (37.6%)	58 (42.0%)	1.201 (0.701–2.059)	0.504	0.522	0.607 (0.258–1.427)	0.252
Sought medical consultation for menopause symptoms^c^	18 (19.4%)	60 (43.5%)	3.205 (1.733–5.927)	< 0.001**	0.621	1.634 (0.620–4.301)	0.321
Sought medical consultation for genitourinary symptoms^c^	13 (14.0%)	64 (46.4%)	5.322 (2.710–10.45)	< 0.001**	0.662	3.401 (1.405–8.232)	0.007**
Sought medical consultation for incontinence^c^	7 (7.5%)	40 (29.0%)	5.013 (2.135–11.77)	< 0.001**	0.607	1.299 (0.382–4.412)	0.675
Hormone therapy^c^	6 (6.5%)	36 (26.1%)	5.116 (2.059–12.71)	< 0.001**	0.598	1.760 (0.481–6.438)	0.393

*Note*: *p < 0.05; **p < 0.01.

^a^ Logistic regression model: all women (*N* = 231); Likelihood ratio test, χ^2^ = 144; *p* < 0.001; Nagelkerke R^2^, 0.626; c‐index, 0.917.

^b^ “Frequency of menopause symptoms” was not included in the multivariate model due to a high correlation with “Distress of menopause symptoms” (Spearman *r*, 0.84).

^c^ Reference groups: Sought medical consultation for menopause symptoms, No; Sought medical consultation for genitourinary symptoms, No; Sought medical consultation for incontinence, No; Hormone therapy, No.

^d^ Mean ± standard deviation or *n* (%).

## 4 Discussion

The findings of this study reveal that the average number of GSM symptoms experienced by participants was 3.64 ± 2.64, with 59.7% reporting distress related to these symptoms. This finding aligns with previous studies indicating that GSM is highly prevalent among postmenopausal women, affecting 50%–84% of this population [12, 13]. However, despite the significant symptom burden, only 33.3% of participants sought medical consultation for GSM, consistent with a previous study reporting low awareness and treatment rates for GSM in Asian populations [19]. In our study, seeking medical consultation for menopause, genitourinary symptoms or incontinence, and hormone therapy correlated positively with the number of genitourinary symptoms and whether they caused distress. The results of multiple ordinal logistic analyses show that the distress level of menopause symptoms significantly correlated with both the numbers of genitourinary symptoms and whether those symptoms caused distress, while distress caused by genitourinary symptoms, BMI > 24.0 kg/m^2^, and seeking medical consultation for incontinence correlated significantly associated with the number of genitourinary symptoms. The number of genitourinary symptoms, number of children, and seeking medical consultation for genitourinary symptoms were significantly associated with distress caused by genitourinary symptoms.

Because GSM is the manifestation of estrogen deficiency, our finding that the level of distress caused by menopause symptoms coincided with the number of genitourinary symptoms and whether they caused distress is reasonable, since all of these factors may correlate with declining estrogen levels. Anatomically, the genital and lower urinary tract share a common embryologic origin in women; both are derived from the same estrogen‐receptor–rich primitive urogenital sinus tissue. During menopause, changes in the expression level of estrogen receptors in these tissues and the decreased serum level of estrogen both contribute to GSM and menopause symptoms. The pathophysiology of GSM includes thinning of the vaginal epithelium and lamina propria, smooth muscle atrophy, decreased blood flow to the vaginal area, loss of collagen and tissue elasticity, and changes in the vaginal flora [24, 25]. Thus, it is predictable that patients who experience a higher level of distress with menopause symptoms also have more severe GSM. This phenomenon is also observed in breast cancer patients treated with aromatase inhibitors. These agents work by blocking the conversion of androgens to estrogens. The magnitude and duration of estrogen deficiency induced by aromatase inhibitors was shown to be associated with the severity of GSM [13]. In clinical practice, healthcare professionals might consider providing the option of additional assessments for GSM for patients who are admitted to the hospital for menopause symptoms, because GSM is more frequently underreported [19] or misconceived as a part of aging than are menopausal symptoms. Chua et al. [19] reported that most patients first heard of GSM through their physician, indicating that awareness on the part of healthcare professionals is important and likely plays a key role in improving GSM management. In addition, a study conducted in Taiwan indicated that more than half (56.4%) of menopausal women who called a menopause hotline preferred to obtain medical information through healthcare professionals, and over 80% of these women reported satisfaction with their medical consultations [10]. Our findings reinforce the need for healthcare professionals to actively inquire about GSM symptoms, even without patient‐initiated discussions. Proactive engagement and culturally sensitive educational programs could help bridge the gap in awareness and treatment for GSM, ultimately improving symptom distress for postmenopausal women. Although GSM is treatable, the Pan‐Asian REVIVE survey reports that 76% of patients were treatment‐naive, underscoring the importance of addressing this issue. This survey also found that 71% of treatment was initiated by healthcare professionals, further indicating the importance of awareness and attitude of healthcare professionals to initiate the evaluation and treatment of GSM. According to the Pan‐Asian REVIVE survey [19], public awareness of GSM is low in Asia compared to the US and Europe; thus, education is needed to address barriers to care such as cultural stigma and lack of knowledge. Community‐based awareness campaigns and engaging in patient‐initiated discussions with healthcare professionals are vital steps in improving the management of GSM symptoms.

Other important factors associated with the number of genitourinary symptoms include BMI >24.0 kg/m^2^, seeking medical consultation for incontinence, and distress caused by genitourinary symptoms. The correlation between higher BMI and GSM may result from weight gain causing pelvic floor dysfunction and altered estrogen metabolism, as proposed by Moral et al. [26] Urinary incontinence often coexists with GSM and is reported to be significantly associated with both stress and mixed incontinence [4, 26]. Estrogen deficiency also causes urinary incontinence. Loss of dermal collagen in dense connective tissue occurs not only in the vagina, but also in the bladder and urethra, leading to incontinence [24]. GSM‐related incontinence is a main cause of recurrent UTI, which significantly affects the quality of life of postmenopausal women. Careful evaluation to clarify the relationship between GSM and incontinence could improve the management of both conditions and avoid the repercussions of inessential antibiotics usage [24]. A key finding of this study is that distress caused by GSM emerged as the strongest predictor of the number of GSM symptoms. This finding is consistent with that of Vivian‐Taylor and Hickey [27], who emphasized that symptom distress, rather than symptom frequency alone, is a critical determinant of reduced quality of life during menopause.

Other important factors associated with distress caused by genitourinary symptoms include the number of genitourinary symptoms, the number of children, and whether medical consultation for genitourinary symptoms was sought. The number of children as a significant predictor of GSM distress is supported previous research by Lambrinoudaki et al. [17], demonstrating a link between parity and pelvic floor dysfunction, which exacerbates GSM symptoms. Similar to the findings of Ruan et al. [18], who reported no relationship between GSM symptoms and age, our study shows no correlation with age.

Our correlation analysis revealed an association between GSM symptoms and hormone therapy use. In our study, 18.2% of women used hormone therapy, which is much lower than the 32% reported in the cohort of Ruan et al. [18]. In comparison, hormone therapy rates ranging from 4% to 16% in European countries are lower than the number reported in our findings [28, 29, 30]. This discrepancy may illustrate the diverse cultural perspectives and medical approaches to managing menopause across different countries. For example, only 25% of the Pan‐Asian REVIVE cohort had specifically discussed their GSM with healthcare professionals, which is much lower than the US‐REVIVE (56%) and EU‐REVIVE (62%) cohorts [19]. This lower rate of seeking care for GSM may result in a lower rate of hormone therapy prescription. Conservative attitudes regarding the discussion of sex‐related issues and the tendency to believe that symptoms are part of a natural aging process that will diminish over time may be the reasons for lower rates of GSM discussion between Asian women and their healthcare providers.

## 5 Limitations

Several limitations should be considered when interpreting these results. A single‐center design and a local convenience sample were used, which may limit the applicability and generalizability of our findings to international populations. The study relied on self‐reported data for both menopause and genitourinary symptoms, introducing potential recall bias or underreporting, particularly regarding symptom frequency and distress levels. Furthermore, the study's cross‐sectional design precludes causal inferences about the relationships between health‐seeking behaviors, menopause, and genitourinary symptoms. Additionally, whether these medical consultations for GSM led to a diagnosis or treatment, and what factors (e.g., physician recommendation or symptom severity) influenced their decision to seek care is unknown. Future longitudinal studies with a more diverse sample and objective clinical measures will improve our understanding.

## 6 Conclusions

In clinical practice, healthcare professionals in Taiwan might consider providing additional assessments for GSM in older women who are admitted to the hospital for other diseases, especially those presenting with a greater number of genitourinary symptoms and experiencing distress caused by genitourinary symptoms, as GSM is frequently underreported yet treatable, and timely identification may improve patient outcomes. To improve the management of GSM in Taiwan, education to increase the awareness of patients, peri‐ and post‐menopausal women, and the community may be the critical step for proactive screening and targeted interventions for GSM.

## Ethics Statement

This study was conducted in accordance with the Declaration of Helsinki and approved by the Institutional Review Board of Kaohsiung Medical University Chung‐Ho Memorial Hospital (protocol code: KMUIRB‐E(II)‐20,220,066 and date of approval: 18 May 2022) for studies involving humans.

## Conflicts of Interest

The authors declare no conflicts of interest.

## Supporting information


**Table S1:** Specific urinary and genital symptoms included in the Genitourinary Symptoms Scale (GSS).

## Data Availability

The data that support the findings of this study are available on request from the corresponding author. The data are not publicly available due to privacy or ethical restrictions.
