# Supplementary material for: Risk Factors Associated With the Number of Symptoms and Distress Caused by Genitourinary Syndrome of Menopause in Taiwanese Women
Source: Kaohsiung J Med Sci. 2025 Dec 17;42(6):e70143. doi: 10.1002/kjm2.70143 (PMC13248810; doi:10.1002/kjm2.70143)
Supplement: Supplementary file 1 — Table S1: Specific urinary and genital symptoms included in the Genitourinary Symptoms Scale (GSS). [file KJM2-42-e70143-s001.docx]

| Table S1. Specific urinary and genital symptoms included in the Genitourinary Symptoms Scale (GSS) | | |
| --- | --- | --- |
|  | n (%) | Mean ± SD |
| 1. Have you had any urine leakage in the past 12 months? | 90 (39.0%) |  |
| 2. Have you used any type of leak-proof product for involuntary urine leakage in the past 12 months? For example: sanitary pads, pads, diapers or nappies. | 39 (16.9%) |  |
| 3. How many times per day, on average, did you urinate when you were awake in the past 12 months? |  | 8.35 ± 2.67 |
| 4. Have you experienced frequent urination in the past 12 months? | 76 (32.9%) |  |
| 5. Have you ever felt a strong urge to urinate without warning in the past 12 months? | 39 (16.9%) |  |
| 6. How many times per night, on average, did you get up at night to urinate in the past 12 months? |  |  |
| 7. Have you experienced one or more interruptions in urination in the past 12 months? | 40 (17.3%) |  |
| 8. Have you needed assistance to start urinating or to keep urinating in the past 12 months? | 15 (6.5%) |  |
| 9. Have you ever felt like you didn't get it all out or couldn't empty your bladder completely after urinating in the past 12 months ? | 63 (27.3%) |  |
| 10. Have you ever felt a weak stream of urine when you urinated in the past 12 months? | 47 (20.3%) |  |
| 11. Have you had any urinary tract infections in the past 12 months? | 80 (34.6%) |  |
| 12. Have you experienced burning in your urethra when you urinate in the past 12 months? | 75 (32.5%) |  |
| 13. Have you had recurrent urinary tract infections in the past 12 months? | 43 (18.6%) |  |
| 14. Have you experienced vaginal dryness in the past 12 months? | 160 (69.3%) |  |
| 15. Have you had any vaginal itching in the past 12 months? | 96 (41.6%) |  |
| 16. Have you had difficulty having sexual intercourse in the past twelve months? | 127 (55%) |  |
| 17. Have you had any vaginal infections in the past 12 months? | 39 (16.9%) |  |
| 18. Have you had recurrent vaginal infections in the past 12 months? | 25 (10.8%) |  |
| 19. Have any of your female family members experienced genitourinary symptoms? | 16 (6.9%) |  |
| 20. To what degree were you bothered by the genitourinary symptoms in the last 12 months? |  | 1.59 ± 0.92 |
| Not at all (0) | 93 (40.3%) |  |
| Mild (1–3) | 83 (35.9%) |  |
| Moderate (4–7) | 40 (17.3%) |  |
| Severe (8–10) | 15 (6.5%) |  |
| 21. Have you ever mentioned your genitourinary symptoms to a health care provider? | 81 (35.1%) |  |
